# Lessons learned from a multimodal sensor-based eHealth approach for treating pediatric obsessive-compulsive disorder

**DOI:** 10.3389/fdgth.2024.1384540

**Published:** 2024-09-24

**Authors:** Carolin S. Klein, Karsten Hollmann, Jan Kühnhausen, Annika K. Alt, Anja Pascher, Lennart Seizer, Jonas Primbs, Winfried Ilg, Annika Thierfelder, Björn Severitt, Helene Passon, Ursula Wörz, Heinrich Lautenbacher, Wolfgang A. Bethge, Johanna Löchner, Martin Holderried, Walter Swoboda, Enkelejda Kasneci, Martin A. Giese, Christian Ernst, Gottfried M. Barth, Annette Conzelmann, Michael Menth, Caterina Gawrilow, Tobias J. Renner

**Affiliations:** ^1^ Department of Child and Adolescent Psychiatry, Psychosomatics and Psychotherapy, University Hospital Tübingen, Tübingen, Germany; ^2^ DZPG (German Center for Mental Health), Tübingen, Germany; ^3^ Department of Computer Science, Communication Networks, University of Tübingen, Tübingen, Germany; ^4^ Hertie Institute for Clinical Brain Research, Section for Computational Sensomotorics, University of Tübingen, Tübingen, Germany; ^5^ ZEISS Vision Science Lab, University of Tübingen, Tübingen, Germany; ^6^ Economics and Management of Social Services, Institute for Health Care and Public Management, University of Hohenheim, Hohenheim, Germany; ^7^ Information Technology Division, University Hospital Tübingen, Tübingen, Germany; ^8^ Center for Clinical Studies Tübingen, University Hospital Tübingen, Tübingen, Germany; ^9^ Department of Medical Development, Process and Quality Management, University Hospital Tübingen, Tübingen, Germany; ^10^ Faculty of Health Management, University of Applied Sciences Neu-Ulm, Neu-Ulm, Germany; ^11^ Department of Educational Sciences, Human-Centered Technologies for Learning, TUM School of Social Sciences and Technology München, München, Germany; ^12^ Department of Psychology (Clinical Psychology II), PFH—Private University of Applied Sciences, Göttingen, Germany; ^13^ Department of Psychology, University of Tübingen, Tübingen, Germany

**Keywords:** usability, sensor technology, obsessive-compulsive disorder, internet-based cognitive behavioral therapy, children and adolescents, exposure

## Abstract

**Introduction:**

The present study investigates the feasibility and usability of a sensor-based eHealth treatment in psychotherapy for pediatric obsessive-compulsive disorder (OCD), and explores the promises and pitfalls of this novel approach. With eHealth interventions, therapy can be delivered in a patient's home environment, leading to a more ecologically valid symptom assessment and access to experts even in rural areas. Furthermore, sensors can help indicate a patient's emotional and physical state during treatment. Finally, using sensors during exposure with response prevention (E/RP) can help individualize therapy and prevent avoidance behavior.

**Methods:**

In this study, we developed and subsequently evaluated a multimodal sensor-based eHealth intervention during 14 video sessions of cognitive-behavioral therapy (CBT) in 20 patients with OCD aged 12-18. During E/RP, we recorded eye movements and gaze direction via eye trackers, and an ECG chest strap captured heart rate (HR) to identify stress responses. Additionally, motion sensors detected approach and avoidance behavior.

**Results:**

The results indicate a promising application of sensor-supported therapy for pediatric OCD, such that the technology was well-accepted by the participants, and the therapeutic relationship was successfully established in the context of internet-based treatment. Patients, their parents, and the therapists all showed high levels of satisfaction with this form of therapy and rated the wearable approach in the home environment as helpful, with fewer OCD symptoms perceived at the end of the treatment.

**Discussion:**

The goal of this study was to gain a better understanding of the psychological and physiological processes that occur in pediatric patients during exposure-based online treatment. In addition, 10 key considerations in preparing and conducting sensor-supported CBT for children and adolescents with OCD are explored at the end of the article. This approach has the potential to overcome limitations in eHealth interventions by allowing the real-time transmission of objective data to therapists, once challenges regarding technical support and hardware and software usability are addressed.

**Clinical Trial Registration:**

www.ClinicalTrials.gov, identifier (NCT05291611).

## 1 Introduction

A total of 14%–20% of children and adolescents are affected by mental illness worldwide (1–3), substantially diminishing their school, social, and health functioning and leading to a considerable socioeconomic burden. Although the demand for psychotherapeutic services has steadily increased in recent years, only about one third of the affected children and young people ever receive psychotherapeutic help (4, 5). Effective approaches are therefore needed to provide appropriate treatment and prevent mental illness from becoming chronic in this population. However, studies on young people's utilization of psychotherapeutic interventions have shown that barriers exist, such as limited understanding of mental health and the desire to manage one's challenges without help (6–8). Shame and fear of social stigma, difficulties opening up to a therapist, and hesitance to trust a stranger were identified as contributing obstacles. In addition, systemic and structural conditions, including high demand for specialist services, limited availability of professional help, long waitlists for therapeutic support, and logistical challenges in accessing child and adolescent mental health services represent additional barriers to therapeutic support.

New technologies are presenting a valuable chance to overcome these barriers, as digital mental health approaches can improve the accessibility, usability, and effectiveness of traditional office-based cognitive behavioral therapy (CBT) by offering an excellent treatment option that does not require travel, avoids fear of stigma, and provides immediate access to experts (9). The fact that today's youth have grown up as digital natives opens up groundbreaking opportunities to supplement therapeutic interventions with digital technologies (e.g., smartphones or smartwatches), making it easy to collect data and deliver CBT interventions to youth and their families. Accordingly, therapeutic exercises can be done at home, which is pivotal for psychiatric disorders such as an obsessive-compulsive disorder (OCD).

An estimated 0.5%–3.6% of children and adolescents suffer from OCD (10, 11), which is characterized by distressing, intrusive thoughts and the use of ritualized actions to relieve anxiety and tension. Although the average age of onset is between 8 and 11 years (12), it often takes several years before affected children and adolescents receive psychotherapeutic treatment (13–15). A shorter duration of illness and an earlier start to treatment appear to be related to better clinical outcomes by preventing treatment resistance (16–18). In some cases, OCD and its related disorders have a severe course that could even lead to suicidal ideation or suicide attempts (19, 20), indicating that individuals with OCD are at greater risk for committing suicide in comparison with the general population (21).

Evidence-based treatments for pediatric OCD have been investigated thoroughly in recent years. Pharmacotherapy with serotonergic antidepressants and cognitive-behavioral therapy have been found to be effective in improving OCD symptoms (22–24). A recent meta-analysis suggested that the combination of pharmacological and psychological treatment shows great efficacy in reducing symptoms in children and adolescents with OCD (25). Weidle et al. (26) provided a detailed overview of the effects of pharmacological treatment for pediatric OCD and comorbid diagnoses from initial medication to relapse prevention (26).

As a psychotherapeutic intervention, CBT is considered the gold standard for treating OCD and should include exposure with response prevention (E/RP) as a core component (27, 28). E/RP requires the person to face their anxious thoughts and feelings without engaging in compulsive behaviors. However, despite its high efficacy, E/RP is rarely performed in practice due to a lack of time and a lack of experienced therapists, especially in remote areas, or disorder-specific symptoms that limit the patient's mobility (29, 30). Internet-based cognitive behavioral therapy (iCBT) can enhance the accessibility and efficacy of psychotherapeutic interventions, thus reducing barriers to conducting exposure sessions. Because the majority of a patient's symptoms occur in the patient's home environment, onsite exposure is essential for the effective treatment of pediatric OCD (31). Particularly in research on OCD, numerous studies have highlighted the relevance of digital interventions for patients suffering from compulsive thoughts and behaviors (32, 33), emphasizing the importance of ecological momentary assessments to capture newly emerging OCD symptoms in daily life (34) and the improvement of the OCD symptom burden through digital or mobile treatments, such as virtual reality exposure sessions for contamination-related compulsions (35). For example, the inclusion of motion sensors revealed changes in sleep patterns in OCD patients (36). In a recent study, episodes of OCD were recognized in the daily lives of adolescents through the use of physiological signals captured by a wearable biosensor on the wrist (37). Especially heart rate (HR) and heart rate variability (HRV) are used as physiological markers of acute and chronic stress responses (38, 39). Under tension, the body releases stress hormones, and HR and blood pressure increase to enhance performance, while HRV simultaneously decreases. Relaxation has exactly the opposite effect, which is why HRV measures reflect autonomic balance and are often used to assess stress responses (40–43).

However, despite positive findings that wearable-assisted therapy can reduce symptoms of mental disorders such as anxiety and OCD (33, 44), the paucity of research on sensor-based methods suggests a significant gap in understanding the limitations and ethical implications of these methods. A review on the use of technology in treatment for anxiety and OCD found that only a quarter of the controlled trials have shown a significant additive effect when using technology-based interventions, despite the benefits demonstrated in symptom reduction (45). The authors emphasized that half of the studies did not report evidence of technology intervention acceptance, whereas the remaining studies did not even prioritize this goal. Therefore, the implementation of customized interventions that are specifically designed to improve technology acceptance will likely produce better outcomes and increased engagement. The scarce research reflects a lack of guidance for researchers and clinicians on how to integrate sensors into their work and a poor understanding of the key considerations in the selection and preparation of a wearable device for use in pediatric therapy and research. In fact, user-centered design and patient engagement through gamification or persuasive technology, along with cross-sector collaboration in program development and data sharing, are considered essential for the successful implementation of digital mental health approaches (46–48). Conversely, poor usability of various mental health apps has been reported, as they are often perceived as difficult or unenjoyable to use (49). Consequently, retention is seen as a crucial challenge for researchers to tackle when developing future interventions (50, 51). For instance, smartphone app attrition rates for depression were found to be 26.2%, which increased by 20% when adjusted for publication bias (47.8%) (52). Similarly, 57.9% of participants in a study of self-guided mobile apps for depressive symptoms never downloaded the study app (53). A review of digital self-help tools revealed reduced adherence in real-world settings, as completion rates for the same tools were much lower (1%–28%) compared with clinical studies (44%–99%) (54). For this reason, patient engagement with digital mental health tools has been found to be a common barrier to the success of eHealth technologies.

In recent years, our team has conducted several studies on internet-based treatment for children and adolescents with OCD. Results have shown that this form of therapy is helpful and highly efficacious (55, 56). At the same time, it has also become apparent that a certain amount of information gets lost due to the small area of the screen through which the therapist and the patient can see each other. This limitation makes it difficult to accurately assess patients’ stress or anxiety responses during exposure sessions. In addition, it is almost impossible for the therapist to examine whether patients are showing avoidance behavior during the sessions, such as trying not to look directly at the symptom-triggering object. Nonetheless, identifying avoidance behavior is crucial for the success of the exposure session. Therefore, the integration of multimodal sensors may be helpful for improving the efficacy of internet-based treatment for OCD.

Consequently, we developed a sensor-based eHealth intervention for pediatric OCD called Smart Sensor Technology in Tele-Psychotherapy for Children and Adolescents with OCD (SSTeP KiZ). In this paper, we report the promises and pitfalls of this unique approach. In a psychotherapeutic trial, 14 CBT sessions were delivered online, supported by multimodal sensors applied to the children. We assessed stress with HR, gaze direction via eye tracker, and repetitive movements with wrist bands. The therapist received real-time feedback from these measures to tailor the session to the patient's individual needs. The treatment was further supported by an ambulatory assessment web application with gamification to encourage the patients to report their experiences with the technical equipment during treatment.

To develop the procedure, we conducted an initial analysis of five OCD-patients from SSTeP KiZ. The findings indicated that it was possible to identify stress and repetitive compulsive behavior as well as avoidance behavior by utilizing these various sensor modalities (57). The technical structure of the system has already been thoroughly described (58). To the best of our knowledge, this study outlines the first attempt to use such a sensor-based approach in pediatric OCD patients and has the potential to provide a better understanding of the processes underlying exposure sessions, with the goal of making this form of therapy more effective in the future. This intervention was designed to evaluate the feasibility of and patient satisfaction with this sensor-based approach. The aim of this study was to investigate the extent to which the entire sensor system with the software and hardware components can be implemented as intended and whether this system can be used during iCBT to treat pediatric OCD. In addition, we explored patients’ ratings of the usability and acceptability of such an approach with different wearables. This included questions related to the patients’ and parents’ user experience with the entire therapy system and treatment adherence as well as their perceptions of the helpfulness of the therapy and the establishment of the therapeutic relationship through iCBT. Furthermore, we examined whether patients completed the questionnaires reliably as a result of the gamification elements that were involved. At the end of this article, we present a checklist we compiled of 10 key points that researchers and clinicians should consider when developing wearable therapy systems and conducting sensor-based treatments in child and adolescent health care.

## 2 Methods

### 2.1 Recruitment and study sample

The main target group of SSTeP KiZ were children and adolescents between 12 and 18 years of age with a primary diagnosis of OCD according to the Diagnostic and Statistical Manual of Mental Disorders, Fifth Edition (59). The sensor system was developed and tested in two pilot groups, one with five children and adolescents with no diagnosis of a mental disorder and the other with 11 children and adolescents with OCD. In the main study, 20 pediatric OCD patients were treated with sensor-supported iCBT, of which the technical components were revised further and made more stable in the first six patients, followed by the other 14 patients. The aim of this study was to assess the feasibility and acceptability of this approach. On the basis of a pilot study and other face-to-face psychotherapy studies, a total of *n* = 20 patients was deemed appropriate for meeting these objectives for this study phase (55, 60). Inclusion criteria were at least one legal guardian and a family home with broadband internet access. If psychiatric comorbidities existed, the comorbid disorder could not have a higher treatment priority than OCD. If medication was used, the dose had to be the same for 6 weeks prior to the diagnostic session and then continued at the same level during the study. The children's living conditions had to be stable to ensure sufficient support for the patients during therapy (e.g., when conducting the video sessions and applying the technical devices). Participants were excluded if they had an IQ below 70, did not speak or understand German, had a psychiatric comorbidity that made participation clinically inappropriate (e.g., eating disorder, major depression), or if they required full inpatient treatment. No other psychotherapeutic treatment was allowed while they were participating in the study. If side effects were reported or if the patients wished, they were able to discontinue treatment at any time, and we assisted them in finding another treatment option.

The average patient age (*n* = 20) was 16.11 (*SD* = 1.64) years, and male and female patients were equally distributed in the sample (55% male). All of them lived in Germany, and German was the primary language spoken at home. The mean age of the mothers (*n* = 19) at the beginning of the study was 47.21 years (*SD* = 5.80), the mean age of the fathers (*n* = 20) was 51.15 years (*SD* = 6.10). With regard to family circumstances, 15 families stated that they all lived together; in three families, the parents were separated; the parents in one family were divorced; and one father was a single parent. Most (92.5%) of the patients’ parents were also born in Germany, 5% came from another European country, and 2.5% from a non-European country. Furthermore, 35% of the parents had a higher education degree (university or equivalent). When the study began, six patients had already been under psychiatric treatment, 10 had received psychotherapeutic treatment, three had experience with exposition therapy, and three had previously been under pharmacological treatment. Of the psychotherapeutic pretreatments, eight were conducted in an ambulatory setting, and two during inpatient treatment. Furthermore, 17 patients showed comorbid diagnoses. Four of the patients lived in rural areas, 13 in medium-sized towns, and three in large cities.

The Ethics Committee of the Medical Faculty at the University of Tübingen, Germany approved the study (877/2020BO1). Participants were recruited through the Department of Child and Adolescent Psychiatry in Tübingen and our advertising campaign on Google AdWords that was linked to our own website. The study team collaborated closely with the University Hospital's corporate communications department. As part of the recruitment process, a promotional film was produced and broadcast on social media. The project was also communicated to local psychiatrists and psychologists in Tübingen as well as to the German OCD Society. We explain the data security concept in more detail in Section 2.7.

### 2.2 Procedure

After participants contacted the study investigators via email or the website registration form, the families who expressed interest were invited by the study team to attend an online video conference to learn more about the study's conditions and the general eligibility requirements. If attendees met the preliminary inclusion criteria, a baseline preassessment was conducted during an in-person appointment with an independent diagnostician at the hospital. Written informed consent and written approval were obtained from all participants and their legal guardians prior to their enrollment. The final decision on participation was then made. After receiving initial training at the Child and Adolescent Psychiatry Department in Tübingen, patients took the sensor system home with them. For therapy sessions, we used the secured video communication platform VidyoConnect®, which was officially recommended by the University Hospital Tübingen for communication with patients due to its high level of security. The video software was installed on an additionally provided Android tablet or alternatively on the family's personal device. During treatment sessions, in which patients used the sensor system themselves at home, we recorded their physiological reactions by measuring HR, movement data, and gaze during the exposure sessions. The psychotherapeutic treatments were carried out by two licensed psychotherapists, each of whom treated 10 patients. Both therapists were trained in CBT for children and adolescents and already had experience with internet-based psychotherapy for OCD from previous clinical trials. We gathered demographic information, treatment history, and family history from our hospital's medical history form. Additionally, patients took an intelligence exam to evaluate their basic cognitive abilities and general fluid intelligence.

**Figure 1:**
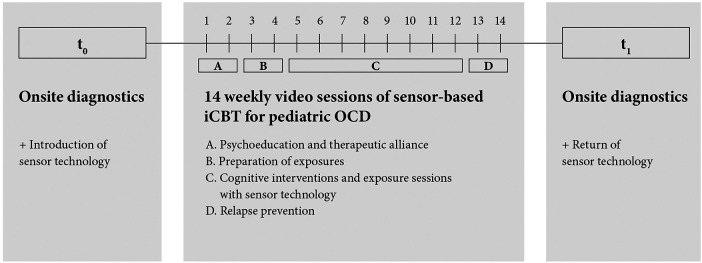
Study design. Figure 1 illustrates the study design and the process of online-based psychotherapy. After an onsite diagnostic assessment and introduction to the use of the sensor system, a 14-week digital CBT consisting of 4 treatment modules took place in the home environment of the patients. At the end of the therapy, the families received a further diagnostic assessment on site and returned the sensor system.

Following the diagnostic evaluation, the main study commenced with the enrolment of the 20 patients who each received 14 weekly video therapy sessions of approximately 90 min each (Figure 1). Based on the German OCD Reference Manual (61), the therapy involved four modules wherein every session comprised a briefing on the objectives, a progress assessment of previous and ongoing exercises, the presentation and practice of new material, and a discussion of the following week's homework. The structure is comparable to the procedure already described in our article on internet-based psychotherapy for children and adolescents with OCD (56).

In this study, we examined the general feasibility of such a sensor-based approach and assessed patient acceptance, compliance, and satisfaction with the different devices and the iCBT treatment. Our aim was to determine whether patients and their families perceived this treatment as helpful and the patient-therapist relationship as stable when the therapy was conducted through teleconferencing. For this reason, we obtained questionnaires at t0 (before treatment) and t1 (after treatment). During the ambulatory assessment, the patients were asked at three distinct points in time about their experiences with the usability of the sensor system, which included eye tracking, movement, heartrate, videocalls, and ambulatory assessment. We evaluated the psychological processes during the exposure sessions in the home environment. Additionally, we analyzed the satisfaction with the ambulatory assessment web application on the basis of patients’ and parents’ responses to the final questionnaires.

The clinical questionnaires that measured changes in symptom severity will be evaluated and described in another paper, as we aimed to focus primarily on technical application and feasibility in the current paper.

### 2.3 Material

#### 2.3.1 Psychological measures

After Session 1, 7, and 14, patients answered a total of 50 questions in the ambulatory assessment with 10 questions for each technical device from the System Usability Scale (SUS) about the handling of the technical equipment (62). For example, they were asked whether the technical devices, the video software, and the web application were easy to use or whether they had encountered difficulties. Questions were answered on a Likert scale ranging from 0 (*I don't agree at all*) to 4 (*I totally agree*). The SUS score can be interpreted on a grading scale, with 90–100 reflecting an A, 80–89 a B, 70–79 a C, and so forth (63). An A can stand for an adjective rating of “Best imaginable,” B for “Excellent,” and C for “Good.”

The aim of the Client Satisfaction Questionnaire-8 (CSQ-8) is to evaluate participants’ perceptions of the value of the service they received at the end of the treatment at t1 (64). The questionnaire recorded patients’ levels of satisfaction with the therapy as well as their intentions to recommend this form of treatment to others in need. Patients answered the questions about the therapy on a 4-point Likert scale, with higher scores indicating higher satisfaction. Overall, total scores can range from 8 to 32.

The Questionnaire for the Evaluation of the Treatment (FBB) at t1 measures how satisfied the patients and their parents were with the treatment and whether the therapy was perceived as helpful (65). The patients were also asked about the therapeutic relationship, that is, whether the therapist was able to understand the patient's situation and build trust. Similarly, questions were answered on a Likert scale with the following anchors: 0 = *Not at all/Never*, 1 = *Hardly/Seldom*, 2 = *Partly/Sometimes*, 3 = *Mainly/Mostly*, and 4 = *Exactly/Always*, with higher scores indicating greater satisfaction with the treatment.

Additionally, we designed implementation and satisfaction questionnaires for the children, the parents, and the therapists at t1. There were also questions about the duration of treatment, the specific content of therapy sessions, and the usability of the technical devices to be answered. The answers were given either on a 4-point Likert scale ranging from 1 to 4 (1 = *I agree*, 2 = *I somewhat agree*, 3 = *I somewhat disagree*, and 4 = *I disagree*) or in free-text fields.

In our study, we used different assessment methods. The Children's Yale-Brown Obsessive-Compulsive Scale [CY-BOCS (66)] is widely used in both clinical and research contexts. It is a semi-structured interview performed by a clinician to examine OCD symptom severity and treatment response. In this trial, the children and adolescents received an onsite diagnostic assessment at the Child and Adolescent Psychiatry Tübingen, in which various clinical questionnaires were used to record the symptoms and severity of the disorder, including the CY-BOCS. The data will be analyzed in a separate manuscript. In the current paper, the evaluation primarily referred to the ambulatory assessment and the final questionnaires administered to patients, parents, and therapists with regard to usability, feasibility, and acceptance of this treatment approach.

After the treatment period, we conducted qualitative focus group interviews with the patients, parents, and therapists. We plan to evaluate these interviews in a different paper.

#### 2.3.2 Physiological measures

**Figure 2:**
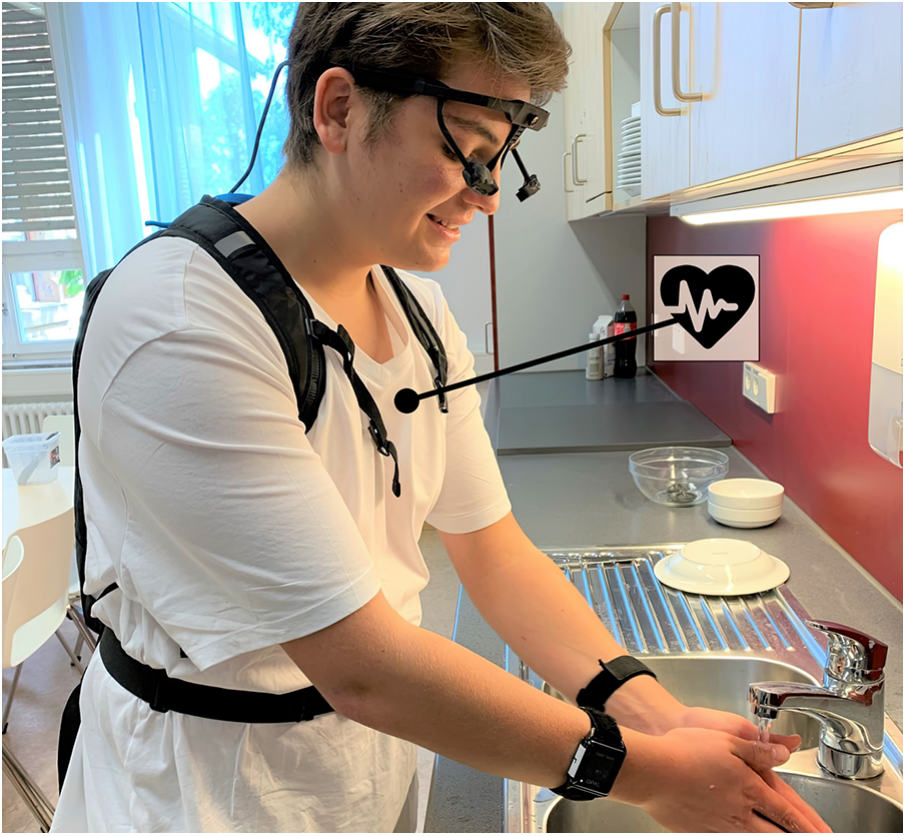
The SSTeP KiZ sensor system. This figure shows the entire sensor system with the ECG chest strap, the motion sensors, and the eye tracking glasses. The tablet with the Aggregator Software, which collects the sensor data, is carried by a small backpack. The University Hospital of Tübingen holds the rights to all images in this paper.

All psychotherapy sessions were delivered online, using an Internet of Medical Things system with multimodal sensor devices, hardware infrastructure, and web applications that combined all modalities for evaluation. In a first analysis of our data, we observed an elevation in HR and a decline in HRV due to heightened stress during exposure sessions (57). The inertial wrist sensors detected an amplification of the energy level from repetitive actions (e.g., walking or hand washing) during periods of increased stress. Moreover, gaze analysis from eye tracking data showed that the patients’ gaze predominantly rested on the object that triggered the stress response. These promising findings highlight the significance of identifying stress and compulsive behavior by utilizing various sensor modalities. The entire sensor system is illustrated in Figure 2.

##### 2.3.2.1 Electrocardiogram (ECG)

HR and HRV are considered physiological markers for acute and chronic stress reactions. A chest-belt ECG sensor (Movesense Sensor HR+, Suunto, Vantaa, Finland, CE-certificated) recorded the electrical activity of the heart at 250 Hz and transferred it to the tablet via Bluetooth Low Energy (BLE). The aggregator software detected R peaks in the signal and extracted RR-intervals for further processing. The average HR was measured in beats per min (BPM), covering an average time window of 30 s. We used the root mean square of successive differences (RMSSD) to display HRV during the sessions. We chose a time window of 5 min to compute the RMSSD in order to adhere to standards and be able to make statements about heightened stress levels (67). The HR oscillations were divided into very-low-frequency (VLF), low-frequency (LF), and high-frequency (HF) bands (67), which were transmitted to the therapist in real time, with the VLF in particular providing information about the patient's state of tension during the sessions.

##### 2.3.2.2 Motion sensor technology

OCD often consists of repetitive actions with a specific movement pattern (e.g., ritualized hand washing or the urge to repeat certain actions several times to reduce anxiety). To record specific movement patterns in patients with compulsions, hand movements were captured by inertial sensors (Opal, APDM Inc., Portland, OR) that were attached to the wrists with Velcro straps. These sensors synchronously recorded acceleration and angular velocity at 128 Hz. Additionally, the acceleration and angular velocity of the trunk were recorded at 6.5 Hz by an inertial sensor in the chest belt, allowing the system to capture patients’ general activity.

##### 2.3.2.3 Data glasses and Eye tracking

Gaze estimations and pupillometry were captured with a custom-built head-mounted eye tracker. Eye movements were recorded with two 320 × 240 px resolution eye cameras along with a 640 × 480 px resolution scene camera, each recording at 30 Hz. Due to its light weight (3D-printed), the device was expected to be well-tolerated by patients. To provide information about the patient's attention, gaze was estimated and displayed in the view of the scene camera (68).

### 2.4 Sensor data analysis

**Figure 3:**
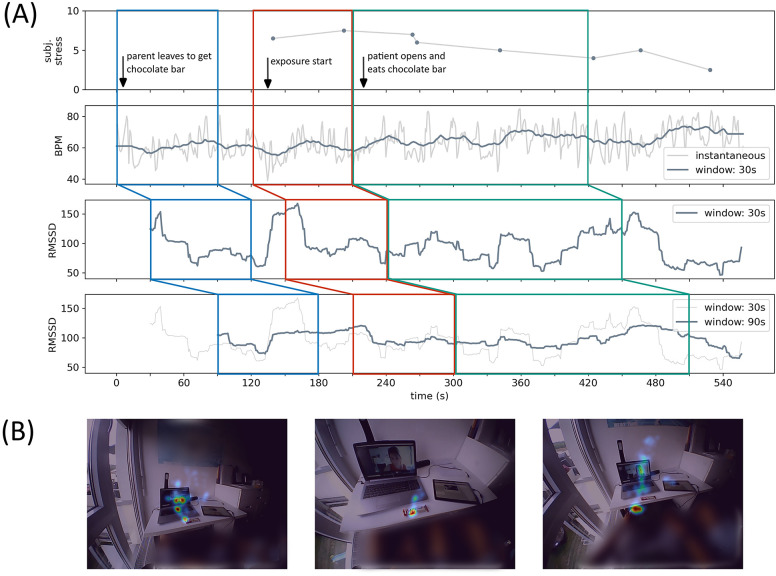
In Figure 3, an exposure session during iCBT is displayed. **(A)** Shows the heart rate data (subjective stress level, beats per minute and heart rate variability) during the exposure. **(B)** Shows the summarized heat maps of estimated gaze points. The patient and his family consented to the presentation of the image material.

To highlight the importance and feasibility of the recorded data, we present a qualitative example for HR and gaze behavior during an exposure session. The patient in Figure 3 showed an obsession with fitness, and in our specific exposure, he ate a bar of chocolate, which he feared would impair his sports career. This exposure was especially suitable as an example because the patient remained seated throughout the exposure, thus minimizing the effects of movement on HR and HRV.

In (Figure 3A), we could observe how HR (middle panel) and HRV (lower panels) coincided with subjective stress level (upper panel). We used the RMSSD in 30 s and 90 s windows to display HRV and indicated the shift in (Figure 3A) with colored boxes. HRV dropped when the parent left the room to get the chocolate bar (red box), reflecting increased stress levels before the onset of the exposure. While there was a brief increase in HRV right before the onset of the exposure, which could be due to spontaneous relief but also due to artefacts, we could see that at the beginning of the exposure, the HRV was again low (red box). It only increased slowly toward the end of the exposure (green box), also reflected in the subjective stress level. This example shows that finding the correct window size and incorporating movement data into the HRV computation to predict stress levels is essential for presenting stress in an online interface.

Figure 3B shows the heat map of the gaze estimations during the two major head poses of the patient in the beginning of the exposure. We were able to observe that the patient's gaze mainly switched between the chocolate bar and the therapist, but most of the patient's attention rested on the chocolate bar. This qualitative example is in line with recent research by our team, where we developed a method for investigating gaze behavior during exposure sessions, and we found preliminary evidence that patients focused more on exposure-related objects with higher stress levels (69).

### 2.5 Tele-psychotherapy and IT architecture

#### 2.5.1 Aggregator device

The aggregator device is the central component of the software that controls the sensors, receives their data, processes the data, and forwards them for streaming or recording. The aggregator software was implemented on a Surface Pro 7 in i7/16GB RAM configuration with Windows 11. Here, the data were prepared to be streamed to the therapist and saved in predefined data formats with synchronized timestamps for post-hoc analysis. During recordings, the patients carried the tablet in a small backpack.

#### 2.5.2 Therapist user interface

**Figure 4:**
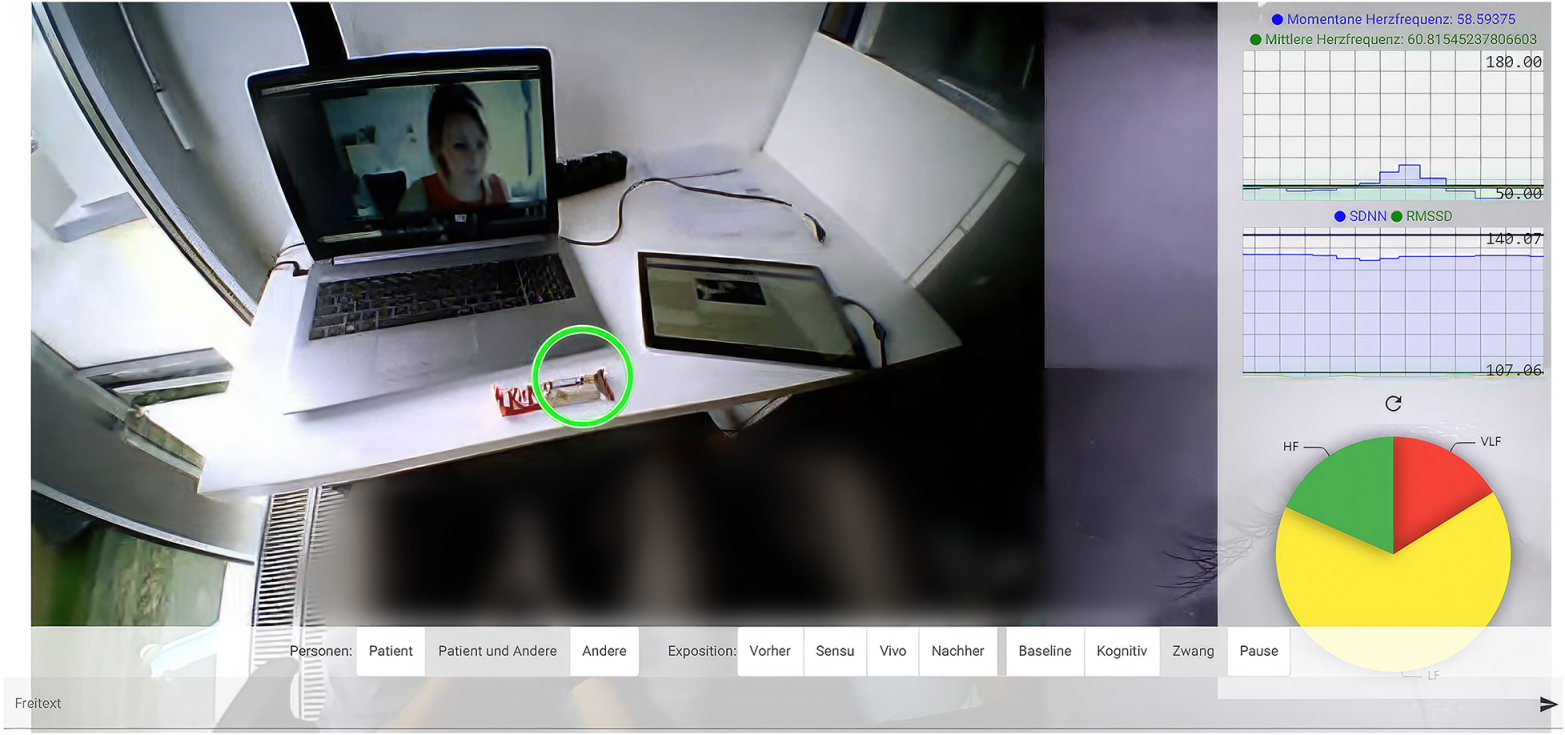
Displays the therapist UI with its different components of gaze estimation, HR and HRV as well as the bar to operate different tags during the sessions. The patient and his family gave their consent to the publication of the image.

The therapist user interface (UI) was a web application that allowed the therapist to access the patient's questionnaires as well as the real-time streaming of the sensor data during the therapy sessions, as illustrated in Figure 4. On the left side of the screen, the patient's field of vision was displayed including the gaze estimation (green circle), pinpointing the object on which the patient was fixating. This helped the therapist to detect avoidance behavior during the exposure exercises and address it if necessary. The patient's HR and HRV were displayed on the right side. At the onset of each therapy session, a brief baseline measurement was taken and included in the diagram as a black line to provide a comparison for increased tension during exposure exercises. In the pie chart at the bottom right, frequency-based metrics of HRV were displayed, showing the ratio of the band powers in HF range (green), LF range (yellow) and VLF range (red), with increased VLF indicating a high level of stress during exposure. Displaying the data in real time should enable the therapist to react to signs of tension, such as elevated HR or possible avoidance behavior, shown in gaze estimations, and to better tailor the session to the individual patient. At the bottom of the screen, the therapist was able to set different tags to document both subjective stress levels and exposure settings to provide context for the evaluation of stress and compulsive actions in the data.

#### 2.5.3 Patient user interface—ambulatory assessment

**Figure 5:**
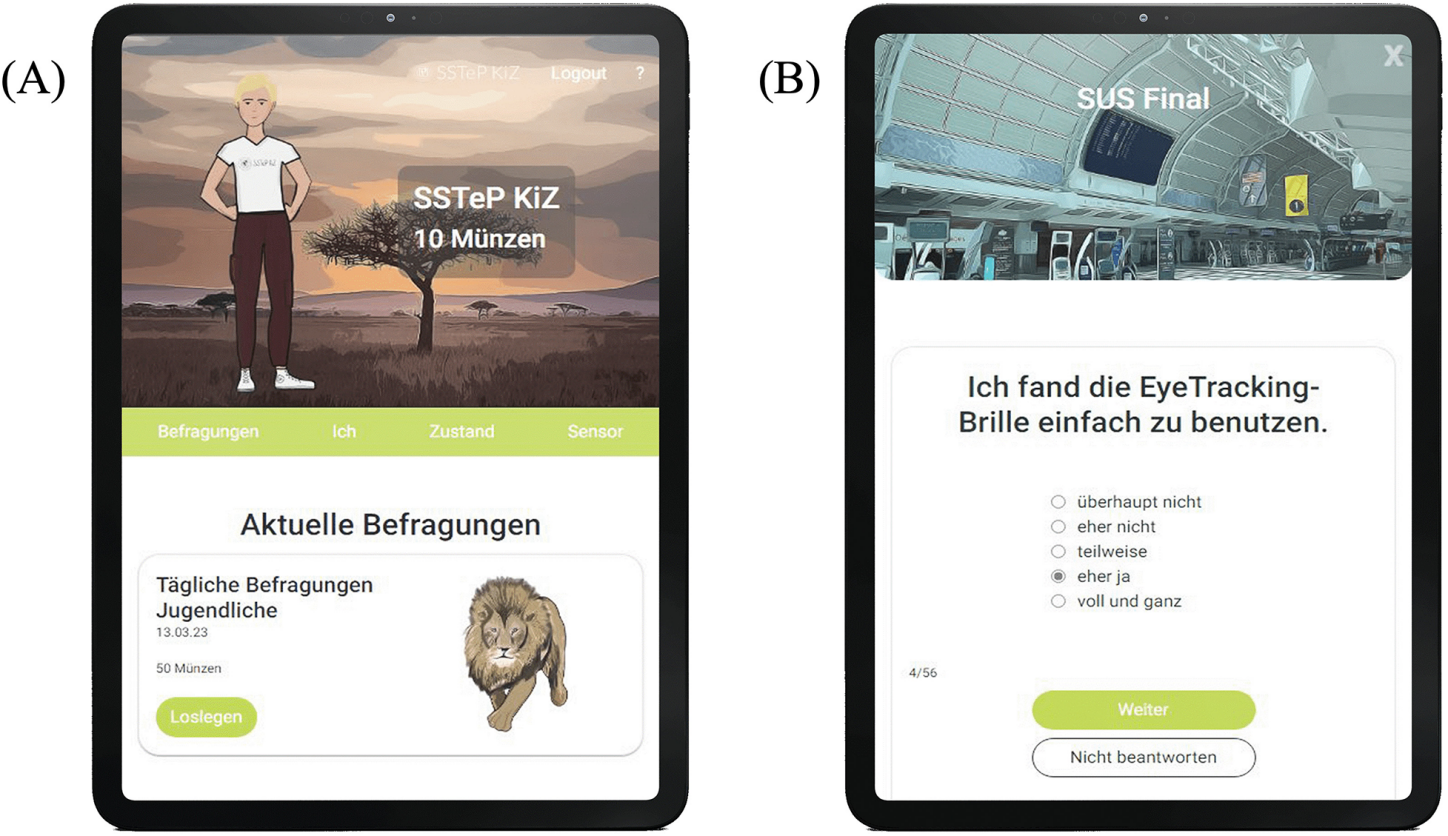
**(A)** Shows the schedule system with the upcoming daily questionnaire. **(B)** displays an example of the questionnaires that were completed by the patients (“I found it easy to use the eye tracking glasses”).

The web application for the patients was developed in cooperation with a software development company (Codext GmbH). Using the app, the children and adolescents were able to evaluate the progress and success of their therapy. The app consisted of a standard questionnaire interface in which an item was presented with one of the common response formats (Likert scale, multiple choice, free text). It was designed to be child- and adolescent-friendly and to encourage patients to consistently complete the daily and weekly questionnaires. For this purpose, a gamification approach was integrated into the web application, where participants could earn coins by completing the questionnaires. The coins could then be used to unlock different continents one by one as destinations on a world map. The app featured a scheduling system that displayed the upcoming questionnaires as illustrated in (Figure 5A). In addition, patients were able to design their individual avatar by buying different clothes and accessories with the coins earned by completing the daily and weekly questionnaires (Figure 5B).

### 2.6 Development period of the therapy system

The system was continuously developed over the course of the 3-year project. The first step of development involved designing the software architecture and integrating all technical system components. This was successfully implemented from May 2020 to the end of September 2021. The system components were then adapted further on the basis of test trials with healthy subjects and the first children and adolescents with OCD. Prototype recordings of symptom-related stress reactions with the complete system were carried out from July 2021 to mid-April 2022. The ergonomics of the technical devices and the software for the patients as well as the reliability of the system were evaluated, revealing a need for optimization in various details. The adaptation of the therapy manual for online psychotherapy for children and adolescents with OCD was completed in August 2021.

After successful completion of the technical trials, during which the equipment (Figure 2) was tested for functionality, the technical system was used in the 14-week treatment of the first six patients with OCD from spring 2022. At the same time, the ambulatory assessment web application was completed from June to the beginning of August 2022, thus allowing patients and parents to digitally answer the questionnaires directly in the app. After successful finalization of the entire technical system and the innovative gamification reinforcement system, the other 14 patients with OCD began the 14-week therapy phase in summer 2022.

### 2.7 Data security concept

The IT security department evaluated the architecture to ensure compliance with medical IT security standards, and the data protection department assessed its adherence to EU-GDPR regulations. All processes were approved by the Ethics Committee of the Medical Faculty Tübingen. Relevant approval from all authorities were obtained. We conducted the study in collaboration with our IT department and were in touch with IT and data security specialists at our clinic. A comprehensive data protection concept was created. The latest data protection regulations were taken into consideration. The data from the ambulatory assessment web application were stored on the internal platform of the University Hospital Tübingen, IMeRa (Integrated Mobile Health Research Platform). The sensor data was saved at an external company in Germany (meerfarbig GmbH/Frankfurt Main). Our data security authorities and the General Data Protection Regulation classified the data as secure. The data were recorded pseudonymously and transmitted in encrypted form, and the research team at the University Hospital Tübingen evaluated the data. The data will be saved for 10 years and will be deleted at the participants’ request. Data were monitored and audited independently of the study's investigators and sponsors. Only the working group of the Department of Child and Adolescent Psychiatry, Psychosomatics and Psychotherapy at the University Hospital of Tübingen, Germany had access to the final data set.

### 2.8 Data analysis

All statistical analyses were performed in R 4.2 (70). Measurements of feasibility, acceptance, and implementation were evaluated descriptively with sample means, SD, and ranges.

## 3 Results

The findings from the questionnaires used to evaluate the treatment and the sensor system are presented separately for the patients (Tables 1, 2), parents (Table 3), and therapists (Table 4).

**Table 1:** Patients’ evaluation of treatment usability and satisfaction.

Item	*M*	*SD*	Range	*N*
System Usability Scale (SUS)				
System Usability Scale—Ambulatory Assessment	83.96	12.01	52.50–98.75	38
System Usability Scale—Video telephony	85.32	11.88	51.45–100	46
System Usability Scale—Eye tracking	83.14	13.89	48.27–98.33	46
System Usability Scale—ECG strap	84.32	17.54	39.17–97.50	46
System Usability Scale—Actigraph	81.52	14.48	55–99.16	46

The results for the SUS are given as the average of the patient mean scores. N, Number of completed questionnaires. For the SUS, questions were answered on a Likert-scale from 0 (I don't agree at all) to 4 (I totally agree).

**Table 2:** Patients’ evaluation of the treatment and sensor system.

Item	*M*	*SD*	Range	*N*	Scale
Therapeutic relationship					
“I was able to trust the therapist.”	1.15	0.49	1–3	20	1 (*agree*)—4 (*disagree*)
“My therapist was interested in me and my problems.”	1.05	0.22	1–2	20	1 (*agree*)—4 (*disagree*)
Ambulatory assessment evaluation					
“I was happy to answer the daily and weekly questionnaires digitally via the app.”	2.14	0.86	1–3	14	1 (*agree*)—4 (*disagree*)
“I liked the design of the app and traveling through the continents.”	2.07	1.14	1–4	14	1 (*agree*)—4 (*disagree*)
“The design of the app as a game motivated me to fill out the questionnaires.”	2.29	1.07	1–4	14	1 (*agree*)—4 (*disagree*)
“I used the app frequently (daily).”	2.14	1.03	1–4	14	1 (*agree*)—4 (*disagree*)
“I enjoyed using the app.”	2.14	1.1	1–4	14	1 (*agree*)—4 (*disagree*)
“I liked the fact that I was able to customize the avatar in the app according to my wishes.”	2	1.24	1–4	14	1 (*agree*)—4 (*disagree*)
Treatment satisfaction					
“If someone I know also had a problem with compulsions, I would recommend the therapy.”	1.4	0.5	1–2	20	1 (*agree*)—4 (*disagree*)
“I liked the fact that the therapy was conducted over the Internet.”	1.8	0.89	1–4	20	1 (*agree*)—4 (*disagree*)
“During the therapy, I thought about the fact that my therapist is only available via the Internet at the moment.”	2.74	1.15	1–4	19	1 (*agree*)—4 (*disagree*)
“I think a therapy with personal contact would have suited me better.”	2.6	1.23	1–4	20	1 (*agree*)—4 (*disagree*)
Perceived helpfulness of treatment					
“My compulsions have diminished compared with before the therapy.”	1.45	0.6	1–3	20	1 (*agree*)—4 (*disagree*)
“My compulsions have increased compared with before the therapy.”	3.45	1	1–4	20	1 (*agree*)—4 (*disagree*)
“The therapy was successful.”	1.55	0.69	1–3	20	1 (*agree*)—4 (*disagree*)

**Table 3:** Parents’ evaluation of the treatment and sensor system.

Item	*M*	*SD*	Range	*N*	Scale
Therapeutic relationship					
“I was able to trust the therapist.”	1.11	0.32	1–2	19	1 (*agree*)—4 (*disagree*)
“The therapist was interested in us and our problems.”	1.03	0.23	1–2	19	1 (*agree*)—4 (*disagree*)
“The therapist was able to help my child.”	1.17	0.38	1–2	18	1 (*agree*)—4 (*disagree*)
Treatment satisfaction					
“If someone I know also had a problem with compulsions, I would recommend this therapy.”	1.66	0.51	1–3	20	1 (*agree*)—4 (*disagree*)
“I liked the fact that the therapy was conducted over the Internet.”	1.35	0.59	1–3	20	1 (*agree*)—4 (*disagree*)
“I think a therapy with personal contact would have suited me better.”	3.25	1.02	1–4	20	1 (*agree*)—4 (*disagree*)
“I would have liked to have answered the weekly questionnaires digitally via an app.”	1.8	1.01	1–4	20	1 (*agree*)—4 (*disagree*)
Perceived helpfulness of treatment					
“My child’s compulsions have diminished compared with before the therapy.”	1.7	0.98	1–4	20	1 (*agree*)—4 (*disagree*)
“My child’s compulsions have increased compared with before the therapy.”	3.6	0.82	1–4	20	1 (*agree*)—4 (*disagree*)
“The therapy was successful.”	1.45	0.61	1–3	20	1 (*agree*)—4 (*disagree*)

**Table 4:** Therapists’ final therapy evaluation.

Item	*M*	*SD*	Range	*N*	Scale
Satisfaction with sensor-based iCBT					
“I liked the fact that the therapy was carried out via the Internet.”	1.3	0.66	1–3	20	1 (*agree*)—4 (*disagree*)
“I think I would have liked a therapy without the Internet, where direct contact with the patient would have been possible, better.”	3.4	0.99	1–4	20	1 (*agree*)—4 (*disagree*)
“I liked the fact that I could see the patient's vital signs and integrate them into the therapy sessions.”	1.7	0.66	1–3	20	1 (*agree*)—4 (*disagree*)
“With the heart rate, I was able to better capture the patients’ tension during the expos.”	2.1	0.79	1–3	19	1 (*agree*)—4 (*disagree*)
“During expos I could easily see where the patient was looking at through the eye tracking.”	1.95	0.83	1–3	20	1 (*agree*)—4 (*disagree*)
“I liked the structure of the therapist UI (streaming, setting tags, etc.).”	2.1	0.55	1–3	20	1 (*agree*)—4 (*disagree*)
“Creating the surveys was easy for me.”	2.05	1.19	1–4	20	1 (*agree*)—4 (*disagree*)
Functionality of technical devices					
“The therapist UI worked well.”	1.85	0.67	1–3	20	1 (*agree*)—4 (*disagree*)
“The transmission of the heart rate (streaming) worked well.”	2.75	0.55	2–4	20	1 (*agree*)—4 (*disagree*)
“The transmission of the eye-tracking camera (streaming) worked well.”	2.55	0.76	1–4	20	1 (*agree*)—4 (*disagree*)
“Connecting the motion sensors worked well.”	2.25	0.44	2–3	20	1 (*agree*)—4 (*disagree*)
“We had to interrupt the therapy or started later because the streaming didn't work.”	1.75	0.85	1–4	20	1 (*agree*)—4 (*disagree*)
Feasibility of sensor-based treatment					
“I found it difficult to monitor the patient's vitals and talk to the patient at the same time.”	2.25	0.44	2–3	20	1 (*agree*)—4 (*disagree*)
“I found it difficult to monitor the patient's heart rate and field of vision during therapy and to talk to the patient at the same time.”	2.25	0.45	2–3	20	1 (*agree*)—4 (*disagree*)
“I found it difficult to set tags during therapy.”	2.85	0.49	2–4	20	1 (*agree*)—4 (*disagree*)
“I found it useful that I received feedback on the emotional state and symptoms from the patients via the app.”	2.45	0.89	1–4	20	1 (*agree*)—4 (*disagree*)
“I used the app data to prepare my sessions.”	2.95	0.61	2–4	20	1 (*agree*)—4 (*disagree*)
“The feedback from the sensory data enabled me to better adapt the session to the respective patient(s).”	2.35	0.67	1–3	20	1 (*agree*)—4 (*disagree*)
Perceived helpfulness of therapy					
“The therapy was successful.”	1.3	0.57	1- 3	20	1 (*agree*)—4 (*disagree*)
“The compulsions of the patient have changed.”	1.25	0.44	1–2	20	1 (*agree*)—4 (*disagree*)
“The patient's compulsions have diminished compared with before the therapy.”	1.4	0.5	1–2	20	1 (*agree*)—4 (*disagree*)
Implementation of treatment procedures					
“The data collected with the help of the app was discussed as intended.”	2.83	0.76	1–4	18	1 (*agree*)—4 (*disagree*)
“The entire technical equipment was used as planned.”	2	0.91	1–3	18	1 (*agree*)—4 (*disagree*)
“The exchange of working materials via online cloud took place as planned.”	1	0	1	18	1 (*agree*)—4 (*disagree*)

The table shows the results of the final questionnaire from the therapists that intended to evaluate feasibility, acceptance, and implementation of the treatment. N, Number of completed questionnaires.

### 3.1 Usability of technical components

The results for the SUS are given as the average of the patient mean scores. According to the SUS, patients rated all technical components on average between 81.52 and 85.32, with the best values for the video conferencing system and the ECG chest strap, followed by the ambulatory assessment, eye tracking and motion sensors. SUS values between 80 and 89 correspond to a rating of B, which stands for excellent usability (63). Table 1 presents detailed results.

### 3.2 Patients’ final questionnaire evaluation

The CSQ-8 was answered on a Likert scale from 1 to 4, with the total score ranging from 8 to 32 and higher scores reflecting a greater level of satisfaction. The patients achieved a mean score of 27.5 (*SD* = 4.02), which suggests a high level of satisfaction with the treatment.

The results of the FBB showed a mean value of 2.95 (*SD* = 0.38), which indicates that the patients were satisfied with this treatment approach.

As shown in Table 2, the final questionnaire included questions about the therapeutic relationship, evaluation of the ambulatory assessment, and satisfaction with and perceived helpfulness of the treatment.

Patients’ ratings indicated that the therapeutic relationship was successfully established during the iCBT and was considered to be trustworthy. Patients appreciated that the app included the option to design a unique avatar. The motivation to complete the questionnaires because of the gamification was slightly above the medium range. Patients were satisfied with the sensor-based treatment approach and found it helpful and successful in the majority of cases.

### 3.3 Parents’ final questionnaire evaluation

The results of the FBB showed a mean value of 3.69 (*SD* = 0.37), which indicates that the parents were highly satisfied with this treatment approach.

The parents completed the final questionnaire on the therapeutic relationship, satisfaction with the treatment, and perceived helpfulness of the approach (Table 3).

The results indicated that a trusting therapeutic relationship was established during the internet-based treatment. The parents were satisfied with the intervention and rated the therapy as successful, such that the parents reported that the patients showed fewer obsessive-compulsive symptoms at the end of the treatment.

### 3.4 Therapists’ final questionnaire evaluation

Like the patients and their parents, therapists answered questions about the feasibility and functionality of the sensor-based approach, satisfaction with the treatment method, and perceived helpfulness and implementation of the treatment, as shown in Table 4.

In the final questionnaire, the therapists reported a high level of satisfaction with the sensor-based approach. They rated seeing the patient's physiological parameters during the session as beneficial, albeit challenging, as more information was added to the therapy session through real-time streaming. Their evaluations of the functionality and implementation of the sensor-based approach were in the good to medium range. The therapists stated that the patients’ compulsions had improved as a result of the treatment.

### 3.5 Data completion

**Figure 6:**
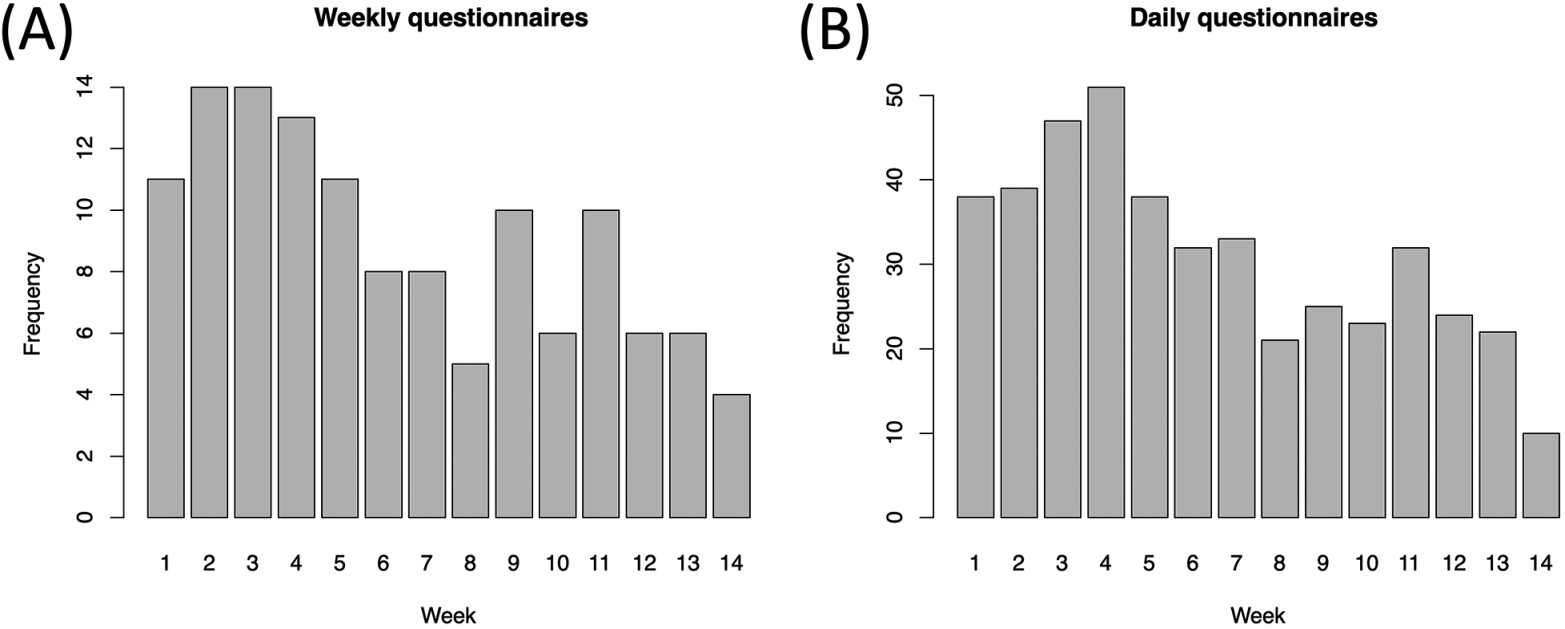
Daily and weekly questionnaires from the patients. Compliance rates as the frequency of completed weekly **(A)** and aggregated daily **(B)** ambulatory questionnaires as a function of weeks into the study.

Compliance with the procedure was evaluated with missing data. Missing rates were observed in the daily (76.7% missing data) and weekly (55.4% missing data) ambulatory assessments, with compliance declining over the course of the study period (Figure 6).

Regarding treatment attrition, all patients and families completed the 14 iCBT sessions with no dropouts.

## 4 Discussion

In this study, we developed and conducted a first test of a multimodal sensor system for children and adolescents with OCD. The purpose was to explore the potential feasibility of such an approach for treating pediatric OCD, including the possibility of conducting therapy sessions and exposures in the home environment supplemented by wearables, and to assess patient, parent, and therapist satisfaction with this kind of treatment. The goal was to investigate the processes during exposure sessions to be able to adapt the treatment more effectively to the specific patients in the future. Generally, obsessive-compulsive symptoms can arise in any environment and can be related to different stimuli. The use of digital technologies and sensor systems could give rise to new OCD symptoms or addictions, particularly in relation to the digital environment (71, 72). In order to prevent a possible addiction to digital health tools, a lot of psychoeducation and family counseling took place during the 14-week cognitive behavioral therapy, during which the family's questions about further symptoms could be answered, and newly developed symptoms could be discussed directly. If OCD symptoms arise in relation to the use of digital therapy methods (e.g., gamification or the individual sensor components), such symptoms should be addressed during the online sessions and exposed if necessary. If it becomes apparent during iCBT that the patient's symptoms are too severe or extend to digital treatment in the form of an addiction, patients should be referred to face-to-face treatment on site. Despite the large number of technology-based studies on mental disorders in recent years (32, 33, 45, 73), there is little guidance on what to consider when developing a sensor-supported approach. In psychological studies, young people are asked for their opinion on digital mental health tools only after the products have already been designed, when there is little time or resources left to make changes (74). In the clinical context, interventions are often developed to actualize the researchers’ intentions rather than to take into consideration what the end users or patients want and how the interventions can be adapted to patients’ daily lives. For this reason, technology-based innovations should be regarded as services for users, rather than just therapeutic products (75). They should be customized to suit users’ lifestyles, interests, and preferences, as user-centered application and design can significantly boost users’ intrinsic motivation to engage with technological devices (76).

### 4.1 Patient ratings

The assessment of SSTeP KiZ showed that patients were accepting of the overall study design and that the sensor system was easy to handle, indicating excellent usability. Patients showed a high level of satisfaction and compliance with this treatment approach. The development of the therapeutic relationship was considered very positive, which was reflected, for example, by the items “I was able to trust the therapist” and “My therapist was interested in me and my problems.” The ambulatory assessment web application also received a moderate to positive evaluation. According to the patients, they appreciated completing the daily and weekly questionnaires in the app and were satisfied with its design. Patients stated that they enjoyed using the app and that they liked customizing the avatar to their own preferences. The feedback on gamification was in a good to medium range. However, despite the positive evaluation of the gamification approach, we found low compliance rates in the daily and weekly ambulatory assessments, with compliance declining over the course of the study period. These results are in line with other studies on digital interventions (54, 77). A possible reason for this decline could be that the patients had to operate various software components: They recorded sensor data through the Aggregator UI and accessed online therapy materials stored on a separate cloud through another platform, while therapy sessions were conducted via the teleconferencing system. Furthermore, patients were instructed to complete daily and weekly questionnaires in the ambulatory assessment web application and were given the opportunity to use gamification to help them complete the questionnaires. Our study's findings suggest that the children and adolescents may have been overwhelmed by the simultaneous operation of multiple technical components and, as a result, answered fewer questionnaires in the ambulatory assessment. The weekly homework tasks that were required of the children and adolescents (e.g., reading worksheets and performing exposure exercises on their own) might also have required cognitive effort and motivation. Another reason for the declining response rate could be that it posed a challenge for patients to frequently enter their own symptoms into an app. The constant reminders of illness were found to have a negative impact on emotional well-being (78) and usually required a significant amount of time and energy, potentially leading to reduced commitment.

The patients rated the treatment as successful and perceived it as helpful, stating that their problems had improved over the course of the treatment and that the OCD symptoms were less severe at the end. All patients attended every session with no dropouts for the duration of the study, thus indicating a high level of motivation to complete the online-based treatment. The exposure exercises were executed as intended in the patients’ home setting where symptoms occur naturally, which should reduce the barrier to performing the exercises in their daily lives at home between sessions and additionally saving both time and travel. Satisfaction with the treatment was reflected, among other things, in the high level of agreement to recommend the treatment to other patients in their environment. Patients liked the fact that the therapy was conducted online. When asked whether they would have preferred therapy with face-to-face contact in the same room, patients tended to disagree. It also hardly seemed to matter that the therapist was available “only” via the Internet.

### 4.2 Parent ratings

The feedback from the parents in the final questionnaire was consistent with the responses from the children and adolescents. The parents also reported a positive relationship with the therapist during the online sessions. They felt that the therapist understood them and their problems, and they indicated a good relationship of trust with the therapist as well as successful cooperation by indicating a high level of agreement with the items “The therapist and I got on well together” and “I was able to trust the therapist.” Parents were very satisfied with the digital treatment approach, which they would recommend to others in their environment. Most parents reported that the fact that the treatment took place online was a great advantage, and they preferred it to face-to-face contact in a room. Like the patients, the parents perceived the treatment as very helpful and successful and rated their children's symptoms as less severe at the end of the therapy. They reported being able to gain a better understanding of their children's problems through the therapeutic discussions.

### 4.3 Therapist ratings

On the final questionnaire completed by the therapists, they reported a high level of satisfaction with the sensor-based approach. They described the real-time transmission of physiological data (e.g., HR recordings and eye tracking) as an advantage because they were able to integrate this feedback into the therapy session. They evaluated the structure of the therapist UI as positive and described it as ease to use when creating surveys for patients. For the functionality of the transmission, the therapists reported some technical difficulties in the reliability of data streaming. In some cases, sessions were delayed due to the technology. Regarding the feasibility of the sensor-based approach, the therapists stated that they sometimes found it challenging to simultaneously observe the patients’ vital signs while setting different tags during the patients’ exposures. The therapists evaluated the feedback from the ambulatory assessment as helpful to a limited extent. Preparing the therapeutic sessions with the help of the app data was also rated as somewhat helpful. The feedback from the sensor data was able to partially help them adapt the sessions to the respective patient. Overall, like the parents and patients, the therapists rated the therapy as successful and noted improvements in patients’ symptoms. Regarding the implementation of the treatment, the therapists stated that all the technical equipment and the cloud at the University Hospital were generally used as planned. Discussions about the data with the help of the app took place to some extent.

### 4.4 Limitations

This feasibility study has some limitations. Since this was the first clinical trial to develop such a complex sensor system, which took a lot of project time and resources, we used a single group design with a small sample size. Despite the participatory design of the ambulatory assessment web application with feedback from test subjects of the same age, adherence was low. This seems to be a common problem in clinical trials, as the focus usually is on the digital CBT approach rather than the gamification application. In a subsequent study, the benefits of completing the regular questionnaires should be further explained to the participants to improve adherence. Furthermore, the exposures were limited to the home setting only because we had to avoid recording uninvolved third parties on the eye tracking videos due to data protection restrictions. In addition, the design of the eye tracker was not entirely suitable for everyday use, as it could not be individually adjusted to the shapes of the patients’ heads and was also quite prominent as a study device. For our upcoming studies, we are planning to develop glasses that can be used in everyday life and are barely noticeable. These glasses should make it possible to implement the exposure sessions outside the home environment, provided that the recording of uninvolved third parties can be done anonymously (e.g., by pixelating their faces). In general, the technology should be further refined as noted by the therapists, and the real-time transmission in the video sessions should be implemented more robustly. Additionally, we assume that our study consisted of a selective patient sample of rather technology-oriented children and adolescents, and that the results may be transferable to standard care to a limited extent.

### 4.5 Key considerations

Our study represents a first attempt to assess and measure patients’ physiological responses to sensor-based iCBT and thus to objectively record stress and anxiety during exposure sessions, a process that can help develop treatment plans and tailor sessions to individuals. This procedure may be able to overcome the limitations of digital interventions and subjective self-report in the future. Despite good treatment results and demonstrated clinical efficacy, sensor-based mental health systems face several issues on their way to being implemented in regular care. Given the lack of extensive research to date, a nuanced assessment of the potential advantages and current challenges of sensor-based treatment is needed. By providing a checklist with 10 key considerations to assess the benefits and barriers of such an approach, our goal was to begin helping researchers and clinicians integrate online mental health technologies into their therapeutic practice and improve the treatment of children and adolescents with OCD (see Table 5). We therefore examined the hurdles of usability and universal design, as such barriers can make wearables challenging to use in therapy, especially for younger populations. We also took a closer look at maintaining adherence and compliance with online-based treatments, as low engagement and attrition in app interventions is considered a critical barrier to the implementation of digital mental health research.

**Table 5:** Checklist of 10 key points to consider before launching a sensor-based therapy approach.

Item	Checklist
I.	**User-centered and age-appropriate design** In addition to innovative and personalized designs, potential users should be involved in participatory design approaches from the very beginning of the development period until the end to ensure that the intervention effectively meets their emotional, motivational, and functional needs.
II.	**Adherence** To maintain patients’ motivation and compliance, persuasive design, behavior change techniques, human support, and in-app mood monitoring are essential, as well as information about the benefits and importance of stable adherence and continuous symptom reporting.
III.	**Attitude toward the use of technology** Extensive education of therapists and participants regarding the benefits and risks of technology-based methods can help overcome skepticism and strengthen the perceived advantages of newly emerging technological interventions.
IV.	**Functionality of the technical devices** Good functionality of the hardware and software components as well as their interaction and the ergonomics should be ensured with the help of regular support from IT staff, and sufficient storage space must be provided for data streaming.
V.	**Simple and easy processes and technology** The decisive factors in sensor-based interventions are the ease of use of the technical system for patients, parents, and therapists; the intuitive design of the user interface to facilitate interaction between the therapist and the families; and the automated processing and presentation of physiological data.
VI.	**Suitable sensors** The technical requirements for the sensors, the evaluation of the data, and the correct application of the technical system to the subjects should be carefully checked in advance to ensure that the data are measured accurately.
VII.	**Interdisciplinary collaboration across various scientific disciplines** Especially for complex studies that include various technical components, a team of experts from different disciplines should be involved and should collaborate closely from the very beginning (e.g., to prepare the design and integration of back-end data acquisition systems into the front-end user interface).
VIII.	**Technical support by and for the therapeutic team** Intense training by IT specialists for patients, parents, and therapists is mandatory (e.g., via detailed script and video instructions on how to operate the different technical devices).
IX.	**Supportive environment for the patients** It can be highly beneficial to involve the parents of younger patients in sensor-supported psychotherapy, as parents can motivate their children to participate in therapy sessions, help them solve technical issues, guide them in the operation of technical devices, and discuss important topics with the therapist if necessary.
X.	**Data protection issues** It is essential to protect sensor data (e.g., eye tracking recordings, speech, and emotion detection) to safeguard against the possibility of uninvolved third parties appearing on the recordings, and the storage of data should be clearly regulated (e.g., on a central research platform).

#### I. Interface design of the technical equipment

Usability can be described as the quality of the user's experience when interacting with a product or system, as well as challenges related to universal design and user-friendliness. Usability also addresses whether the application successfully serves its intended purpose (79). Digital technologies for children's and adolescents’ mental health should be age-appropriate to ensure effective engagement across a range of ages (80). Research on youth online interactions shows that this population frequently chooses online technology for socializing, planning activities, and expressing affection (81). Therefore, it is crucial to use a human-centered approach that addresses users’ emotional, motivational, and functional needs in the most effective way (48, 74). However, in order to use an evidence-based approach and ensure the same systematic conditions for all participants, digital mental health tools often only present excerpts from therapy manuals provided to all participants at a specific time, an approach that does not support young people's desire for independence and control and therefore diminishes the sense of personalized and dynamic interventions (82). Such outdated practices lead to a mismatch between the existing needs of the users of these products and the issues typically addressed by online interventions from clinical research (83). A recent review article on patients’ views of digital health products identified patient empowerment, self-management, and personalization as the most important factors in patients’ use of such tools (84). For this reason, the entire development and design process of patient-centered digital mental health tools should support participatory design approaches by involving potential users and thus taking full account of the needs of the target group (85). In our study, we obtained feedback from potential users on the design, user experience, and structure of the gamification at various points in the project. The young participants responded positively to the illustrations and the overall interface design. Due to time constraints, we only had limited opportunity to adapt the app before incorporating it into the project. As a conclusion, innovative and personalized designs are crucial for digital mental health products to attain recognition for their relevance to users and effectively enhance the mental well-being of this generation in the long run.

#### II. Adherence

A previous study investigated the extent to which intervention design qualities predict adherence among users of real-world behavioral eHealth interventions (86). The study found that among six quality ratings, therapeutic persuasiveness, defined as incorporating convincing design and behavior change techniques, was the most robust predictor of adherence. In addition, the offer of human support (e.g., therapeutic reinforcement and fostering feelings of connection) has been shown to benefit users of internet interventions (46, 87, 88). Therefore, whenever feasible, the opportunity to consistently interact with a therapist, an avatar, or even a chatbot should be offered. Such an opportunity to interact could be a promising approach for future studies, as it provides a platform for interaction between sessions and encourages patients to continue treatment. In our case, we incorporated gamification, but the web application itself did not contain any behavior change support.

In a small feasibility study, four different devices were given to healthy vs. chronically ill participants (89). The results showed that rates of overall adherence to the use of devices were 16% for participants with chronic illnesses and 76% for the healthy control group. Furthermore, device adherence decreased among all participants throughout the trial, as also observed in our SSTeP KiZ study. This finding is consistent with other articles that have found low long-term participation in mental health applications (90, 91). Strategies that have improved retention include providing human feedback and the use of in-app mood monitoring. Also, participation was reported to be significantly higher in studies offering financial incentives (77); however, such incentives may be ethically controversial in the therapeutic context of patient populations. Studies have shown improved motivation and compliance in psychiatric patients, if they recognize an advantage gained through the specific intervention (92). For this reason, it is crucial to explain to patients exactly why it is important to report their symptoms and the benefits such adherence offers in the study context.

#### III. Practitioners’ and patients’ attitudes toward technology

Although we live in an era when new technological advances are constantly generated and delivered, many clinicians and patients alike lack knowledge of current digital mental health interventions and the therapeutic advantages they provide (47, 93). One of the reasons why telemedicine and sensor-based treatments are rarely used in psychotherapeutic practice is clinicians’ skepticism toward technology-based interventions. In a survey of 515 psychiatrists, 79.6% of participants rated the various technology systems studied as risky, suggesting a lack of knowledge about these new technologies (94). The majority assessed wrist-worn sensor data as moderately (46.8%) to minimally (34.9%) acceptable in terms of risk. To improve the implementation of sensor-based techniques in psychotherapy, it is important to help reduce such barriers for practitioners. Also interesting is the finding that patients perceived online-based methods as much more satisfying than their therapists did (95). For patients, the relationship with the therapist and the establishment of the therapeutic alliance during the online conversations were viewed much more positively, whereas psychologists perceived the technology as an element that limited the therapeutic process (96), especially with children (97). They found it difficult to establish trustworthy cooperation comprising understanding, empathy, and warmth during online sessions and to generate a complete picture of the patient's state due to the lack of visual data and other information (95). Sensor-based methods may help overcome such challenges of online therapy by accurately capturing facial and verbal expressions, gestures, gaze, and tension to provide more precise patient information and interaction during therapy sessions. In our study, therapists perceived the insights they gained through the technical devices as advantageous, despite the technical issues. Therefore, in our opinion, it is important to encourage practitioners and patients to engage with new technical interventions and devices, even if they have concerns about digital mental health approaches.

#### IV. Functionality of technical devices

In this paper, we already discussed how internet interventions can overcome obstacles to psychotherapeutic services, such as travel time, a lack of access to experts, and social stigmatization. At the same time, digital mental health approaches can also create barriers: Patients might feel uncomfortable engaging with online technologies or may have trouble operating them (74). In some cases, children have even reported fear of the devices (44). For this reason, it is important that the devices are comfortable to wear, making it easier for patients to use them. The hardware and software components as well as their interaction should be sufficiently tested and tried out in both the laboratory and field, and the functionality should be continuously improved. Attention should be paid to ensuring that patients have a sufficiently stable broadband internet connection, which should be tested prior to their participation. In our study, there were repeated brief outages in the connection or disruptions in the transmission of data in real time because the internet connection was too weak. Many technical resources were needed in the study. During the exposure sessions, a member of the IT staff was present on a regular basis to start the data transfer remotely on the patients’ computers. Furthermore, streaming in real time transports large amounts of data. To adequately transfer and store data for extended periods and to allow for later evaluation, sufficient storage space must be ensured. The initial six patients began the study without ambulatory assessment because the app was still under construction when the project began and was susceptible to errors that might have compromised the user's experience.

#### V. Easy and simple use of technical devices and processes

To ensure user-centeredness, the system must be easy to operate by children, young people and therapists (47). Besides an appealing design, secure navigation through the displays as well as comprehensible instructions and straightforward operation are important. Despite patients’ and their parents’ positive evaluations of our entire sensor-based therapy system, it was evident that the integration of wearable technology into online CBT demands a considerable amount of cognitive effort, not only for participants but also for practitioners. The therapists described “keeping an eye on everything at the same time” as sometimes exhausting, especially because different screens were used. The video session was held in the video program, the physiological data was transferred in the therapist UI, and the therapy materials were shared with the patients on another screen in the cloud of the University Hospital Tübingen. At the same time, the therapist was expected to give instructions on how to proceed during the CBT sessions and establish a sustainable relationship with the patient. Thus, therapists had to consider significantly more factors than in natural face-to-face encounters with the patient. A vital improvement to this approach could be to create a single screen to map all therapeutic interactions, including the transfer of physiological data, sharing of therapy materials, and video settings. Automated recognition of anxiety or stress and avoidance behavior presented to the therapist during sessions would be valuable. Such automatization could facilitate the processing of various types of information. We also discussed in advance whether patients should be able to see their own data and how the parameters might change on the basis of their reactions (98). In SSTeP KiZ, we decided not to grant patients access to graphical representations of their physiological data, as sharing these data could affect their behavior.

#### VI. Are the sensors suitable for measuring what is needed?

Several key considerations are recommended when selecting a wearable device for research purposes (98). First, a thorough literature review should be conducted to assess whether the specific device and the metric of interest have been validated for the population at hand (99). The appropriate sampling frequency and interval should be carefully considered to capture meaningful changes in the specific dimension. At the same time, especially with high-resolution video, it is important not to set the sampling rate too high, as it will consume a lot of processing power and will require a lot of storage. Wearable devices should be evaluated to ensure that they provide accurate measurements of the data they are assessing, as studies suggest that certain devices may underestimate physiological measures, such as HR (100). In this sense, recording a baseline measurement in the resting state is of vital importance. To prevent measurement errors, it is crucial to properly attach the chest strap and avoid slippage. Particularly when it comes to detecting stress responses, not only is it important that the device itself accurately collects the data, but also that researchers are able to precisely interpret the data (98). Instead of using a commercial app to detect HR and HRV, we developed an aggregator software with a patient and therapist UI that allowed us to independently access all the raw data for accurate data analysis. However, this approach required a significant amount of time during the development process. In order to better understand the physiological data collected, participants should answer a few brief questions after a stress response is detected to verify the interpretation of the data (101). This technique could be significantly beneficial to patients undertaking exposure exercises without a therapist. Therefore, various tags were incorporated into our patient interface to enable patients to provide additional information about their level of tension while they were exposed to OCD triggers.

#### VII. Interdisciplinary collaboration among various scientific disciplines

Especially for studies that include various technical components, a team of experts from different disciplines should be involved from the very beginning. In our study, important milestones of the project were identified in regular meetings and implemented in cooperation with the different fields. The team consisted of specialists from, for example, medicine, psychotherapy and psychiatry, health economics, IT and software development, and specialized areas of sensor technology, such as eye tracking and movement. After development, the entire system was first tested within the team for weaknesses and errors, a process that can take a lot of time and effort for everyone involved. Especially at the beginning of the project, designers and IT specialists or engineers should be in close contact in order to integrate the back-end data acquisition system into the front-end user interface (74). Such a therapy system could be designed so that patients’ user behavior can be identified and quantified automatically and in real time. Such a process would be relevant for research on users’ interactions with the system (e.g., which parts of the system they used the most, where they spent the most time). In our study, professionals from different fields of research showed great commitment to developing an appealing design and process for children and adolescents. Our IT experts played a significant role in creating user interfaces for therapists and patients, as well as in transferring sensor data. The software required constant updating to ensure that it is operated smoothly across all technological devices. In this trial, we configured a script that checks for updates at every restart and installs them directly. Technical experts should work closely with clinicians throughout the entire process to continuously update the software.

#### VIII. Technical Support By and For the Therapeutic Team

The use of a multimodal sensor system in therapy with children and adolescents requires a high level of technical support. Therapists carry out most of this support directly during the therapy sessions, as was the case in our study. However, the services of an IT specialist are often needed to resolve connection problems between the therapist and patient, to provide support for managing the different wearables, or to address configuration issues. In some cases, it was necessary for our IT team to connect to the study tablets via remote access in order to provide direct assistance. Streaming in real time can significantly reduce battery life. Thus, it is critical to ensure that tablets and wearables are fully charged before therapy sessions so that exercises can be performed effectively. The therapists in our study were trained by IT specialists so that they could comprehend technical processes and implement them in the therapy. Before the therapy began, all patients and parents were given detailed instructions on how to operate the technical devices at our clinic. During the treatment period, the IT specialists also repeatedly joined in at the beginning of the therapy sessions to provide technical assistance. We prepared a detailed script for the patients with illustrations that described the handling of the technical devices in detail. Participants in our study suggested recording individual explanatory videos for both software and hardware components to facilitate their application and operation in the home setting.

#### IX. A supportive social environment in therapeutic and technological processes

The findings on parental involvement in CBT for youth anxiety indicate that it can be an effective intervention, although it has not been found to directly improve outcomes (102). Recently published systematic reviews have shown no significant difference between CBT treatments with and without parental involvement (103). However, age should be taken into consideration as younger children may require more parental support than older adolescents. Family-based CBT was found to be a well-established treatment for younger children (104). Parent CBT and combination treatments involving group CBT for parents and group CBT for children are also perceived as potentially efficacious treatments. Exposure-based CBT has been shown to be effective when parents are involved. In this regard, involving the parents of younger patients can be highly beneficial in sensory-based psychotherapy. Parents can motivate their children to participate in the therapy sessions, help them with technical issues, or guide them in the operation of technical devices. Furthermore, the practitioner can clarify important issues with the parents, and the parents themselves can get involved in the therapy if necessary. Communication between parents and the therapist helps parents acquire information about their child's clinical condition and how they can support therapy objectives from home. At the same time, it is obvious that unstable living conditions can interfere with the implementation of therapy. Nevertheless, some young individuals desire greater autonomy and would prefer not to involve their parents in therapy. Therefore, it is crucial to discuss these preferences well in advance with the patients and their parents.

#### X. Data storage and protection

When handling patient data, it is crucial to thoroughly address all data protection concerns (48). The vulnerable nature of the content, particularly when it comes to sensor data recorded in the patients’ homes, warrants careful attention. The protection of eye tracking recordings, speech, and emotion detection is essential to safeguard against the possibility of uninvolved third parties appearing on the recordings. In our trial, we prioritized participant confidentiality and privacy during the entire process. The tablets were fortified by IT security professionals against various physical and cyber assaults. We installed a parental control filter for websites so that the children and adolescents were not exposed to inappropriate material. The parents and patients signed consent forms agreeing that the patients would use the tablets only for therapeutic purposes. We provided all patients and their parents with a patient information sheet and a declaration of consent for the storage of their data. Participants in the study were given the choice to withdraw their participation and halt the processing of data at any time. To gain sufficient storage capacity, we used a central cloud server to process, integrate, and store data from the various sensors. The study data was synchronized with the Nextcloud server via a TLS-protected line (HTTPS) from which the therapists could retrieve the data. We turned off the Nextcloud server after the study period for cost-related reasons. In hindsight, a central platform, such as a research center, would have been a better option for data storage.

## 5 Conclusion

The objective of this feasibility study was to report on an innovative sensor-supported therapy system for children and adolescents affected by OCD and to deliver practical recommendations for the use of such a therapy approach from a technical and clinical perspective. The study's findings indicate that the new technology provides an improved understanding of the fundamental mechanisms involved in exposure-based psychotherapy, particularly in the patients’ domestic surroundings. This first attempt showed a high level of satisfaction and usability of the sensor-based therapy approach, although the technical processes need to be further stabilized for reliable application in therapeutic settings. A gap still remains between research and practice, especially when it comes to children and adolescents (105). For this reason, it can take up to 17 years for evidence-based methods to be implemented in clinical practice (106). Bridging this gap requires practitioners to become engaged and to overcome their hesitations regarding online psychotherapy for both themselves and their patients. Furthermore, to ensure successful implementation, it is crucial for researchers to assess efficacy and effectiveness in real-life settings rather than solely in controlled laboratory environments (107). We anticipate that the findings, in conjunction with the key considerations outlined in our checklist, will encourage additional investigations in this area and will enhance the treatment of pediatric OCD as well as other disorders.

## Data Availability

The raw data supporting the conclusions of this article will be made available by the authors, without undue reservation.
